# Quality Testing of Difficult-to-Make Prescription Pharmaceutical Products Marketed in the US

**DOI:** 10.1001/jamanetworkopen.2020.13920

**Published:** 2020-08-24

**Authors:** Adam C. Fisher, Alex Viehmann, Melika Ashtiani, Richard L. Friedman, Lucinda Buhse, Michael Kopcha, Janet Woodcock

**Affiliations:** 1 Center for Drug Evaluation and Research, Food and Drug Administration, Silver Spring, Maryland

## Abstract

**Importance:**

Health care practitioners and patients must have information to support their confidence in the quality of prescription pharmaceuticals.

**Objective:**

To determine whether there were clear and substantive differences in major quality attributes between difficult-to-make solid oral dosage form pharmaceutical products marketed in the US.

**Design, Setting, and Participants:**

This quality improvement study analyzed US Food and Drug Administration–collected samples of 252 drug products marketed in the US and manufactured in the US, Canada, Europe, India, and the rest of Asia. These drug products were immediate-release solid oral dosage forms considered difficult to make on the basis of product quality history. This sampling included 35 innovator and 217 generic drug samples manufactured by 46 different firms containing 17 different active ingredients. Statistical analysis was performed from February to November 2019.

**Main Outcomes and Measures:**

All products were tested within their shelf life on the basis of the legally recognized tests of the US Pharmacopeia for the major quality attributes of dosage unit uniformity and dissolution. These tests measure dosage consistency and drug release, respectively. The consistency of either attribute was used to calculate a process performance index to describe the variability in manufacturing.

**Results:**

All 252 drug product samples met the US market standards for dosage unit uniformity and dissolution, although the process performance index (Ppk) for dissolution fell below the level of 4-sigma capability (ie, <1 error per 1600) for 11 different manufacturers and for generics in 4 of 5 regions, including the US. As part of a retrospective analysis, manufacturers performing above the median Ppk for either dissolution or dosage unit uniformity submitted fewer product quality defect reports (mean field alert reports of 0.22 and 0.63, respectively) than those falling at or below the median Ppk for these attributes (mean field alert reports of 2.1 and 1.7, respectively).

**Conclusions and Relevance:**

All samples met the US market standards for dosage unit uniformity and dissolution, indicating acceptability for use by patients regardless of manufacturer or region. To our knowledge, this is the largest sampling study of pharmaceutical manufacturers for the US market and these data provide objective insight into the quality of prescription drugs with high manufacturing risks.

## Key Points

**Question:**

Are there substantive differences in the major quality attributes of difficult-to-make pharmaceutical products manufactured throughout the world and marketed in the US?

**Findings:**

In this quality improvement study, all of 252 drug product samples met the US market standards for the major quality attributes of dosage unit uniformity and dissolution, although there was evidence of differences in the consistency of these attributes among regions and manufacturers.

**Meaning:**

These findings suggest that difficult-to-make prescription pharmaceuticals marketed in the US consistently meet quality standards even when manufactured outside the US.

## Teaser

This quality improvement study examines quality testing of prescription pharmaceutical products that are manufactured in various regions of the world and marketed in the US.

## Introduction

Health care practitioners (HCPs) and their patients have little objective insight into the quality of prescription pharmaceuticals in the US. This can lead to biases and misunderstandings that affect clinical use. In the US, the Food and Drug Administration (FDA) is responsible for ensuring the quality of legally marketed pharmaceutical products while also protecting confidential and proprietary information about these products. The FDA’s regulatory responsibilities grew as the supply chain for US pharmaceutical products rapidly expanded because of globalization. Recent reports^1,2,3^ have questioned the overall quality of the US prescription drug supply, particularly for generic drugs and international manufacturing. Some HCPs have been vocally skeptical of the quality of certain drug products, seemingly using limited or anecdotal evidence.^1,4^ A recent survey^5^ of HCPs conducted by the FDA and WebMD showed biases associated with drug quality. For example, 74% of HCPs either did not know or were not sure whether they believed that drugs manufactured outside the US and legally marketed in the US adhere to strict manufacturing standards and regulations required by the FDA. Patient perception also impacts clinical behavior. Seventy-three percent of HCPs reported the reason they write dispense-as-written for prescription brand-name drugs is because patients specifically request them to do so. Hospitals even make purchasing decisions based on limited public knowledge of pharmaceutical quality problems.^6^ Although quality is often viewed as a manufacturing issue, the impact on patient use based on the perception of quality is a clinical issue.

To conduct what is, to our knowledge, the largest ever comparative test of the quality attributes of prescription drug products legally marketed in the US, the FDA procured 322 different samples of solid oral drug products manufactured in the US, Canada, Europe, India, and the rest of Asia. Although no feasible sampling study is representative of the entire state of pharmaceutical manufacturing, these data provide objective insight into the quality of some of the most difficult-to-make prescription drugs in the US market.

## Methods

### Sampling Strategy

**Figure 1.**
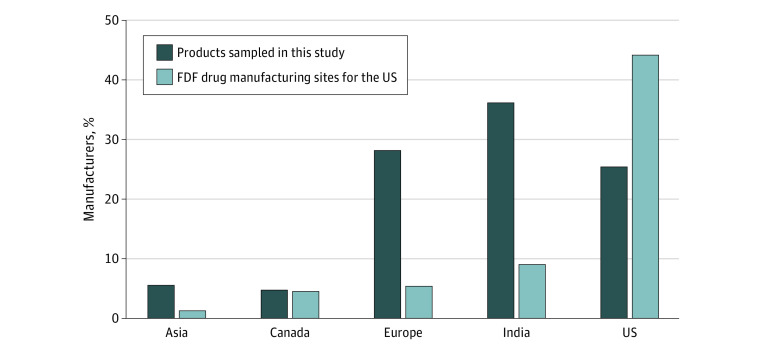
Percentage of the 252 Drug Product Samples and Finished Dosage Form (FDF) Manufacturing Sites Registered With the US Food and Drug Administration by Region

This study was not submitted to an institutional review board for approval because it did not involve human patients and used publicly available data, in accordance with 45 CFR §46. Of 322 samples, 252 were immediate-release products and the focus of this study. The sampling focused on difficult-to-make products that had therapeutic significance and a product quality history of recalls, consumer complaints, MedWatch reports, or field alert reports (FARs) prior to the initiation of this study. This included 35 innovator and 217 generic drug samples containing 17 different active ingredients manufactured by 46 different firms (Table). The number of finished dosage form manufacturing facilities from each region (US, Canada, Europe, India, and the rest of Asia) was gathered from the FDA site inventory as of September 30, 2019. Facilities in India accounted for 36% of the sampled products but represented 9% of the total finished dosage form manufacturing sites for the US market (Figure 1). FDA field investigators collected most drug products directly from manufacturing firms or from drugs imported to the US. Whenever possible, samples were collected from at least 3 lots for each product. Samples were collected within specified expiration dating, allowing time for product testing. When samples could not be obtained from the firm, drug products were purchased from a pharmacy or distributor. All samples were procured prior to the end of 2015.

**Table.** Immediate-Release Drug Products Sampled

Products and ingredients
Alprazolam tablet
Amoxicillin capsule and tablet
Amoxicillin and clavulanate potassium tablet
Citalopram tablet
Ezetimibe and simvastatin tablet
Hydralazine tablet
Metformin tablet
Metoprolol tartrate tablet
Metoprolol tartrate and hydrochlorothiazide tablet
Metronidazole tablet
Oxcarbazepine tablet
Pioglitazone and metformin tablet
Pravastatin tablet
Propafenone tablet
Propranolol tablet
Propranolol and hydrochlorothiazide tablet
Simvastatin tablet
Venlafaxine tablet

Samples of brand and generic drugs were taken from 46 anonymous manufacturers located in 5 regions (Canada, Europe, US, India, and the rest of Asia). Lower limits of dissolution were 70%, 75%, 80%, and 85%.

### Quality Attributes

Samples were evaluated for the critical attributes of (1) dosage unit uniformity, a measure of the individual content of the active ingredient in each capsule or tablet and an indicator of dosage consistency, and (2) dissolution, a measure of drug release in solution and a reflection of the in vivo drug release profile.^7^ These attributes are established pharmaceutical quality indicators.^8,9^ The constancy of either attribute can be used to gauge the manufacturer’s process performance via the process performance index (Ppk), which describes the long-term variation relative to the midpoint of a manufacturing distribution.^10^ The US Pharmacopeia (USP) requirement for dosage uniformity is that the amount of drug substance must fall within 75% to 125% of the label claim.^11^ The USP specifies methods for dissolution testing and compendial limits differ according to the drug product and the active ingredient.

### Testing Methods

For dosage unit uniformity, all samples were analyzed with the method described elsewhere by the USP.^11^ However, regardless of L1 testing results, all 30 units from each lot were tested and used for analysis. If a USP method did not apply to a given product or ingredient, the method described in the approved regulatory marketing application was used.

For dissolution, 12 units were tested from each sampled lot following the USP method specific to that drug product and active ingredient. If testing results dictated that stage 2 testing was required per USP, each cohort was further tested as required. All values were reported as means of the tested ingredients as a percentage of their expected value (ie, as claimed in the label).

### Retrospective Analysis

Final FARs received by the FDA for each sampled product from each manufacturer were counted from October 1, 2015, to September 30, 2018. For the purpose of calculations, manufacturers were considered those with a unique FDA Establishment Identifier Number, and products were based on application numbers. Each active ingredient in products with multiple active ingredients was assigned the number of FARs for the product. The mean number of FARs submitted was calculated for manufacturers performing below or at the median and above the median for dosage unit uniformity or dissolution Ppk.

### Statistical Analysis

The variability of a quality attribute can provide information on the overall manufacturing process performance. The Ppk describes the long-term variation compared with the midpoint of a manufacturing distribution. Although the criticality of pharmaceutical manufacturing may justify capabilities exceeding 6-sigma, many industries use 4-sigma capability (Ppk = 1.33) as a manufacturing performance benchmark, indicating that more than 99.994% of manufacturing process outputs are expected to fall within the specified limits.^12^ As Ppk depends on the distance from specification limits, Ppk values were calculated by separating samples into 4 cohorts of active ingredients that share a lower dissolution boundary to allow comparison based on manufacturer or region (US, Canada, Europe, India, and the rest of Asia). The 95% CIs for dosage unit uniformity and dissolution were calculated using the *t*-distribution with *n* - 1 *df*. Statistical analyses were performed from February to November 2019 using R statistical software version 3.5.1 (R Project for Statistical Computing). See eAppendix in the Supplement for additional statistical information.

## Results

**Figure 2.**
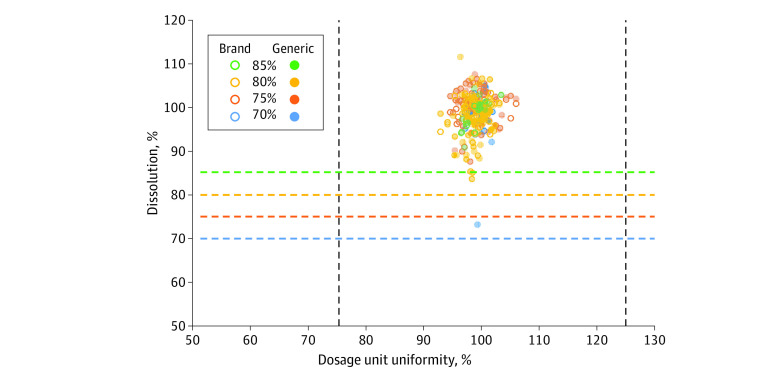
Dissolution vs Dosage Unit Uniformity for Each Active Ingredient in Each Sample Dissolution bounds depicted by dashed vertical lines.

When tested for dosage unit uniformity (Figure 2), all of the 277 active ingredient samples in the 252 drug products sampled met the USP criteria of 75% to 125% of the label claim. All active ingredient samples easily met these criteria as the lowest and highest 95% CI bounds for dosage unit uniformity of all samples were 89.4% and 106.4% of the label claim.

The active ingredients sampled here have lower dissolution bounds of 70%, 75%, 80%, or 85% that define the 4 cohorts of the study.^13^ When tested for dissolution (Figure 2), all samples met the appropriate USP criteria. The lowest 95% CI bound for all active ingredients in each dissolution cohort was also above the lower dissolution bound for that cohort (72.2%, 84.5%, 81.9%, and 88.3%, respectively). For 5 active ingredients, the lower 99% tolerance intervals fell below the lower dissolution bounds: (1) amoxicillin in amoxicillin and clavulanate potassium tablets, (2) hydrochlorothiazide in metoprolol tartrate and hydrochlorothiazide tablets, (3) pravastatin in pravastatin tablets, (4) simvastatin in simvastatin tablets (including simvastatin and niacin extended release tablets), and (5) venlafaxine in venlafaxine tablets.

**Figure 3.**
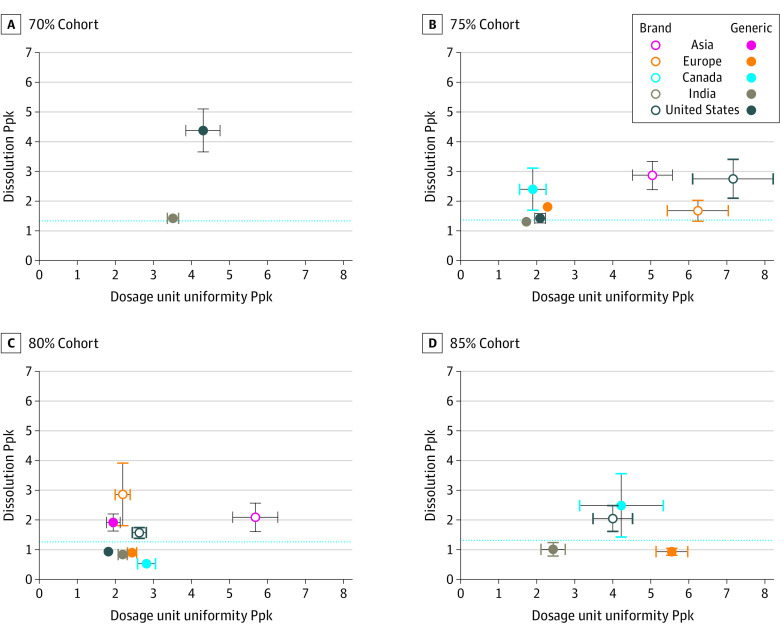
Process Performance Indices (Ppks) for Dissolution vs Dosage Unit Uniformity for Each Cohort Horizontal dashed lines represent 4-sigma benchmark Ppk of 1.33 for dissolution performance. Solid vertical and horizontal lines and error bars show 95% CIs.

When sorting by region, all cohorts for all regions had dosage unit uniformity Ppks (Figure 3) above 1.73, exceeding 5-sigma capability. By contrast, variability in dissolution performance led to 4 of the 5 regions having a generics cohort fall below a 4-sigma benchmark Ppk of 1.33 (Figure 3): India (75%, 80%, and 85% cohorts), Canada (80% cohort), Europe (80% and 85% cohorts), and the US (80% cohort). Conversely, no brand-name drug cohorts fell below a dissolution Ppk of 1.60 for any region. Although all drugs met the tested standards, brand-name drugs generally had higher process performance for dissolution.

**Figure 4.**
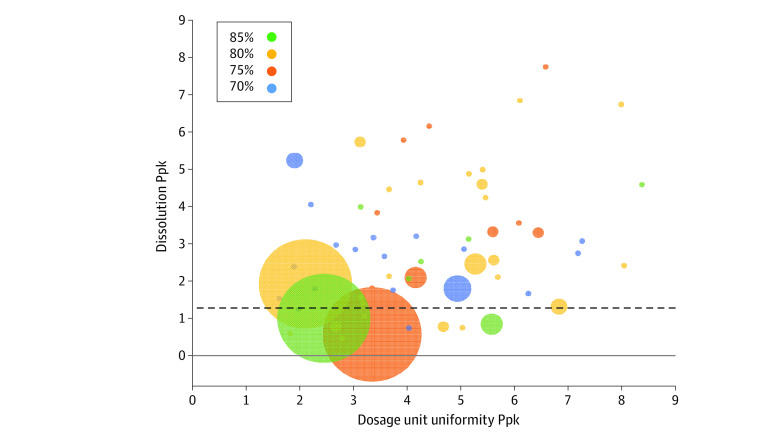
Process Performance Indices (Ppks) for Dissolution vs Dosage Unit Uniformity for Each Manufacturer Horizontal dashed line represents 4-sigma benchmark Ppk of 1.33 for dissolution performance. N = the number of field alert reports (FARs) received from each manufacturer for the sampled products after the end of the sampling period. The diameters of the bubbles are scaled based on N+1 (ie, manufacturers with zero FARs appear as the smallest circles).

When sorting by manufacturer, all cohorts for all manufacturers had dosage unit uniformity Ppk above 1.63, exceeding 4-sigma capability. Variability in dissolution performance (Figure 4) led to 11 manufacturers having a cohort fall below a 4-sigma benchmark Ppk of 1.33 (Figure 4), with 4 of those manufacturers receiving warning letters from the FDA. The FDA receives FARs from applicants when they receive information concerning substantial quality problems with distributed drug products. As part of a retrospective analysis, manufacturers performing above the median Ppk for either dissolution (2.6) or dosage unit uniformity (4.0) reported fewer product quality defect reports (mean FARs of 0.22 and 0.63, respectively) than those falling at or below the median Ppk for these attributes (mean FARs of 2.1 and 1.7, respectively) (see eFigure in the Supplement). The association of FARs with a manufacturer’s process capability is visualized in Figure 4, with FARs depicted as the diameter of bubbles using cohort dissolution and dosage unit uniformity Ppk values for each manufacturer; the largest bubbles fall close to the origin of the graph.

## Discussion

Quality is the basis for consistently safe and effective drugs. Problems with manufacturing and quality issues contribute to nearly two-thirds of all drug shortages.^14^ The FDA regularly reports publicly on data generated in support of drug marketing applications (eg, clinical trials or bioequivalence studies).^15^ Here, samples of difficult-to-make drug products were procured and tested directly by the FDA. Drug sampling studies are needed because such studies in other countries, especially developing countries, have shown serious issues with drug quality,^16,17,18,19^ often associated with counterfeiting.^20^ Beyond this study, the FDA regularly samples drugs for surveillance and other reasons and takes action when occasionally finding substandard products from all regions. Although it is not possible to obtain a statistically representative sample of the entire US pharmaceutical market, this is, to our knowledge, the largest known sampling study of its kind.

Several conclusions potentially drawn from these data run counter to established biases. For example, although there has been skepticism about the quality of generic drugs manufactured for the US market in international facilities, the Ppks suggest that US manufacturers made some of the generic drugs sampled here (Figure 3) with comparable, if not slightly lower, consistency than Indian and Asian manufacturers (Figure 3). This suggests that geographic region alone is not a reliable indicator of quality.

This study highlights additional opportunities for regulatory outreach and scrutiny. For example, it is widely thought that the pharmaceutical manufacturing industry operates between 2 to 3 sigma, well below other manufacturing industries.^12^ Indeed, 11 manufacturers had dissolution cohorts falling below 4-sigma manufacturing associated with dissolution (Figure 4). It is interesting to note that 4 of these 11 manufacturers have received FDA warning letters since the end of the sampling period, all associated with concerns about the reliability of data but unrelated to this study. Retrospective analysis suggests that process performance data may have some predictive power as manufacturers performing above median Ppks reported fewer quality problems (FARs) to the FDA after the end of the sampling period.

With regard to the specific drug products sampled, it was observed that much of the variability in dissolution across all manufacturers was associated with 5 active ingredients for which the lower 99% tolerance intervals fell below the lower dissolution bounds: (1) amoxicillin in amoxicillin and clavulanate potassium tablets, (2) hydrochlorothiazide in metoprolol tartrate and hydrochlorothiazide tablets, (3) pravastatin in pravastatin tablets, (4) simvastatin in simvastatin tablets (including simvastatin and niacin extended release tablets), and (5) venlafaxine in venlafaxine tablets. Although all samples of these products met market standards supporting their continued clinical use, this finding suggests that these products, regardless of who manufactures them, may warrant additional scrutiny. Indeed, the FDA is monitoring stability data for these products submitted in the annual reports of approved drug applications.

It is important to note that a predicted error in pharmaceutical manufacturing does not necessarily mean a substandard product would make it to the market, as release testing should lead to its rejection by the manufacturer. Furthermore, the sampling for this study was completed prior to several of the FDA’s efforts to improve drug quality.^21,22,23,24^ This study focused on prescription pharmaceuticals legally marketed in the US. The safety of counterfeit drugs or drugs purchased online cannot be guaranteed and may present consumers with a health risk from substandard products.^25^ New technologies such as blockchain may improve the ability for regulatory agencies to fight against low-quality, or even counterfeit, drugs.^26^

### Limitations

This study has some limitations. Any conclusions must be drawn only on the samples and attributes represented in this study. As all samples met the tested quality standards, there were no concerns surrounding quality associated with these major attributes or with continued patient use of any product sampled. True Ppks may be higher or lower than calculated here on the basis of additional sampling, especially for individual manufacturers.

## Conclusions

In this quality improvement study, all 252 samples of difficult-to-make prescription pharmaceuticals met the US market standards for dosage unit uniformity and dissolution, indicating acceptability for use by patients regardless of manufacturer or region. These data may provide objective insight into the quality of prescription drugs with high manufacturing risks.

## Notes

Supplement. **eAppendix.** Statistical Methods **eFigure.** Mean FARs Submitted to FDA by Manufacturer After Sampling Period **eReferences.**
