# Supplementary material for: Quality Testing of Difficult-to-Make Prescription Pharmaceutical Products Marketed in the US
Source: JAMA Netw Open. 2020 Aug 24;3(8):e2013920. doi: 10.1001/jamanetworkopen.2020.13920 (PMC7445591; doi:10.1001/jamanetworkopen.2020.13920)
Supplement: Supplement. — eAppendix. Statistical Methods eFigure. Mean FARs Submitted to FDA by Manufacturer After Sampling Period eReferences. [file jamanetwopen-3-e2013920-s001.pdf]

Supplementary Online Content

Fisher AC, Viehmann A, Ashtiani M, et al. Quality testing of difficult-to-make prescription pharmaceutical products marketed in the US. *JAMA Netw Open*. 2020;3(8):e2013920. doi:10.1001/jamanetworkopen.2020.13920

eAppendix. Statistical Methods

eFigure. Mean FARs Submitted to FDA by Manufacturer After Sampling Period

eReferences

This supplementary material has been provided by the authors to give readers additional information about their work.

© 2020 Fisher AC et al. *JAMA Network Open*.

eAppendix. Statistical Methods

95% confidence intervals for dosage unit uniformity and dissolution were calculated using the CI function from the RMISC package in R statistical software as:

$$\bar{x} \pm \frac{t^* \, \sigma}{\sqrt{n}}$$

Where $\bar{x}$ is the mean, σ is the standard deviation, n is the sample size, and t^* is the 1 – (α/2) critical value for the t distribution with $n - 1$ degrees of freedom wherein α = 0.5.

The process performance index (Ppk) utilizes the long-term sigma estimate (i.e., overall variability) calculated as:

$$\hat{P}_{pk} = \min\left[\frac{USl - \bar{X}}{3\sigma}, \frac{\bar{X} - LSL}{3\sigma}\right]$$

Where USl is the upper specification limit, LSL is the lower specification limit, $\bar{X}$ is the mean of the process, and σ is the standard deviation of the process. In all cases, the USP limits for these attributes are used as the USl and LSL. Average Ppk values were calculated for each dissolution cohort sharing a lower specification limit (i.e., 70, 75, 80, 85%) to enable comparison between values.[1]

95% confidence intervals for Ppk were calculated as:

$$Lower\ bound = \hat{P}_{pk} - Z_{1-\alpha/2}\sqrt{\frac{1}{\left(\frac{Toler}{2}\right)^2 N} + \frac{\hat{P}_{pk}^2}{2v}}$$

$$Upper\ bound = \hat{P}_{pk} + Z_{1-\alpha/2}\sqrt{\frac{1}{\left(\frac{Toler}{2}\right)^2 N} + \frac{\hat{P}_{pk}^2}{2v}}$$

Where N is the total number of observations, α is alpha for the confidence level, v is the degrees of freedom ($N - 1$), $Toler$ is the multiplier of the sigma tolerance (6 as the default value), and $Z_{1-\alpha/2}$ is the 1 – (α/2) percentile from the standard normal distribution.[2]

© 2020 Fisher AC et al. *JAMA Network Open.*

eFigure. Mean FARs Submitted to FDA by Manufacturer After Sampling Period

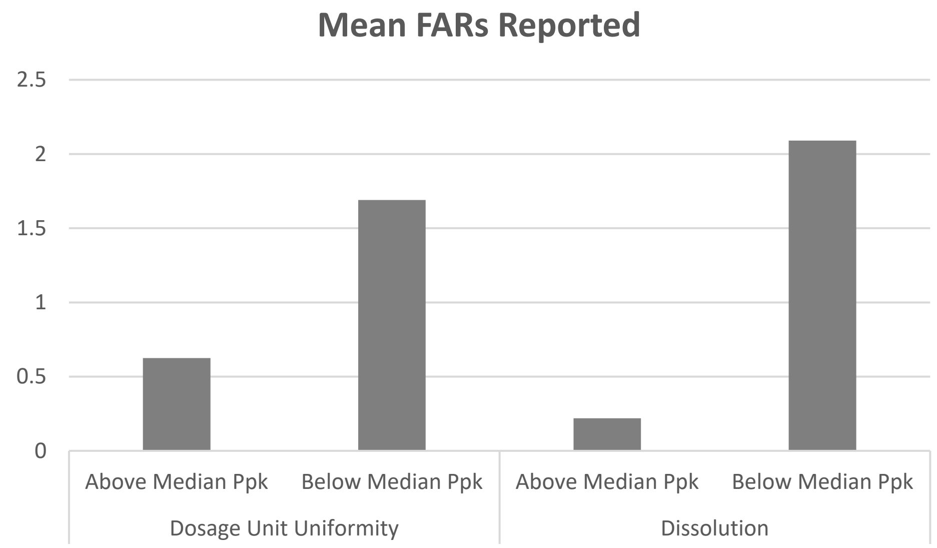

eReferences

[1]Bothe DR. A capability index for multiple process streams. *Quality Engineering.* 1999;11(4):613-618.

[2]ASTM. Standard Practice for Process and Measurement Capability Indices. In. *Designation: E2281 − 08a*: ASTM International West Conshohocken, PA; 2012.

© 2020 Fisher AC et al. *JAMA Network Open.*
